# A Case of Nonocclusive Mesenteric Ischemia During Bioradiotherapy With Cetuximab

**DOI:** 10.7759/cureus.57229

**Published:** 2024-03-29

**Authors:** Nobuyuki Kuribayashi, Norihiko Tokuzen, Hiroyuki Goda, Satoshi Hino, Daisuke Uchida

**Affiliations:** 1 Department of Oral and Maxillofacial Surgery, Ehime University Graduate School of Medicine, Toon, JPN

**Keywords:** oral squamous cell carcinoma, bioradiotherapy, cetuximab, septic shock, nonocclusive mesenteric ischemia

## Abstract

Nonocclusive mesenteric ischemia (NOMI) causes mesenteric ischemia and intestinal necrosis despite the absence of organic obstruction, such as thrombi and emboli in mesenteric blood vessels, and it has an extremely poor prognosis. We report a case of NOMI developed during bioradiotherapy (BRT) with cetuximab for cervical lymph node metastasis of tongue cancer. The patient was a 73-year-old man who underwent right radical neck dissection for neck lymph node metastasis after tongue cancer surgery. Postoperatively, the patient received BRT with cetuximab. On the 34th day after BRT, the patient had abdominal distension and a decreased level of consciousness. Contrast-enhanced computed tomography revealed mesenteric ischemia without thrombi and extensive intestinal emphysema. The patient was diagnosed with NOMI. Furthermore, he had septic shock and was treated with vasopressors and antibacterial agents; however, the condition of the patient did not improve, and he died on the same day.

## Introduction

Nonocclusive mesenteric ischemia (NOMI) is a disease in which mesenteric ischemia leads to intestinal necrosis although there is no organic obstruction of mesenteric vessels; it has a poor prognosis [1]. The risk factors for NOMI include old age, dialysis, arrhythmia, cardiac failure, and unstable circulatory dynamics, such as shock [2]; however, to the best of our knowledge, no studies have focused on NOMI occurring during bioradiotherapy (BRT) with cetuximab. Here, we report a case of NOMI that occurred during BRT.

## Case presentation

The patient, a 73-year-old male, first presented in April 2015 with a chief complaint of pain on the right lingual border. His past medical history included hypertension, chronic renal failure, and hyperuricemia, while both parents had a history of hypertension, and his mother had a malignant tumor. The patient had a significant habit history of smoking (20 cigarettes/day from 25 to 62 years old) and alcohol consumption (100 mL of sake/day, daily). The initial assessment in April 2015 revealed a small 6 × 4 mm bump and a surrounding white spot on the right lingual border. Subsequent to an excisional biopsy (oral intraepithelial neoplasia /carcinoma in situ: OIN/CIS) performed in May 2015 under general anesthesia, a mass formation was observed in the same area in August 2018. This led to a partial tongue resection for squamous cell carcinoma (SCC), with pathological tumor (pT)1, grade (G)1, according to the Yamamoto-Kohama (YK) classification 3. Unfortunately, recurrence prompted another partial tongue resection in July 2019 (SCC, pT3, G3, YK-3). During follow-up in October 2019, enlargement of the right cervical lymph node was noted. Blood tests showed a creatinine level of 2.06 mg/dL and an estimated glomerular filtration rate (eGFR) of 25.7 mL/min/1.73 m^2^. The patient had a continuous decreased renal function since the initial visit, but no other abnormal findings were found.

**Figure 1:**
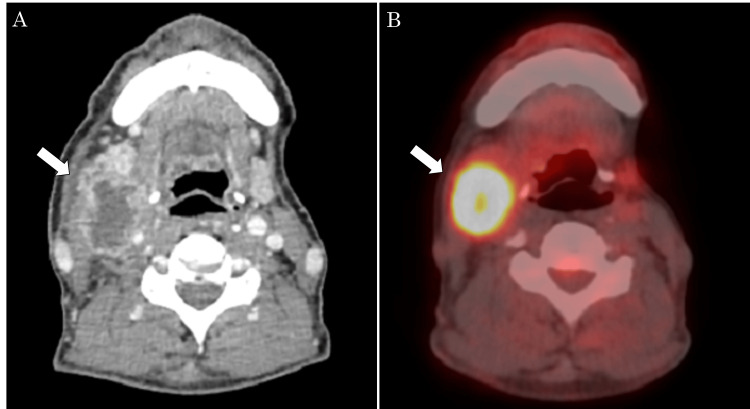
Contrast-enhanced CT and PET-CT images at the time of cervical lymph node metastasis. (A) Lymph nodes with the internal hypoabsorption region with surrounding contrast enhancement were found in the right neck, compressing the internal jugular vein. (B) FDG accumulation was observed in the right cervical lymph node (SUVmax = 15.5). CT: computed tomography, PET-CT: positron emission tomography–computed tomography, FDG: fludeoxyglucose F18

Echocardiography showed no abnormal findings. In terms of general health, the patient exhibited a height of 166 cm and a weight of 54 kg, indicating good nutritional condition. Blood tests revealed a continuous decrease in renal function since the initial visit, with a creatinine level of 2.06 mg/dL and an eGFR of 25.7 mL/min/1.73 m^2^. Echocardiography showed no abnormal findings. Upon examination, the right superior internal jugular lymph node was palpated as a 30 × 25 mm elastic, firm, immobile lymph node. Intraoral findings during follow-up did not show evidence of recurrence, despite the presence of surgical scars. Imaging studies, including contrast-enhanced computed tomography (CT) and positron emission tomography (PET)-CT, revealed a lymph node (33 × 25 mm) with contrast enhancement around the right cervical lymph node (Figure 1A), along with fluorine-18 deoxyglucose accumulation (maximum standard unit value of 15.5) in the same lymph node (Figure 1B).

The patient was admitted to our hospital in October 2019 and underwent tracheostomy and right-sided radical neck dissection under general anesthesia. In the neck dissection, metastatic lymph nodes were adherent to the internal jugular vein and common carotid artery. The internal jugular vein was resected, and the part adhering to the common carotid artery was resected using a scalpel as much as possible. Histopathological examination showed extracapsular invasion only in the lymph node, two metastases in the superior internal deep cervical lymph nodes, and one metastasis in the middle internal deep cervical lymph node. On the 27th postoperative day, contrast-enhanced CT revealed a lesion with contrast enhancement on the margins in the right lateral neck and right lateral mediastinum and a nodular shadow in the right lower lobe of the lung, suggesting right lateral neck recurrence, right lateral mediastinal lymph node metastasis, and right lateral lung metastasis. After consulting with the thoracic surgeon, they diagnosed the lesions as amenable to curative resection based on imaging findings. On the 29th postoperative day, the patient underwent thoracoscopic partial resection of the right lower lobe and tumor resection of the right mediastinum under general anesthesia at the Department of Respiratory Surgery of our hospital.

**Figure 2:**
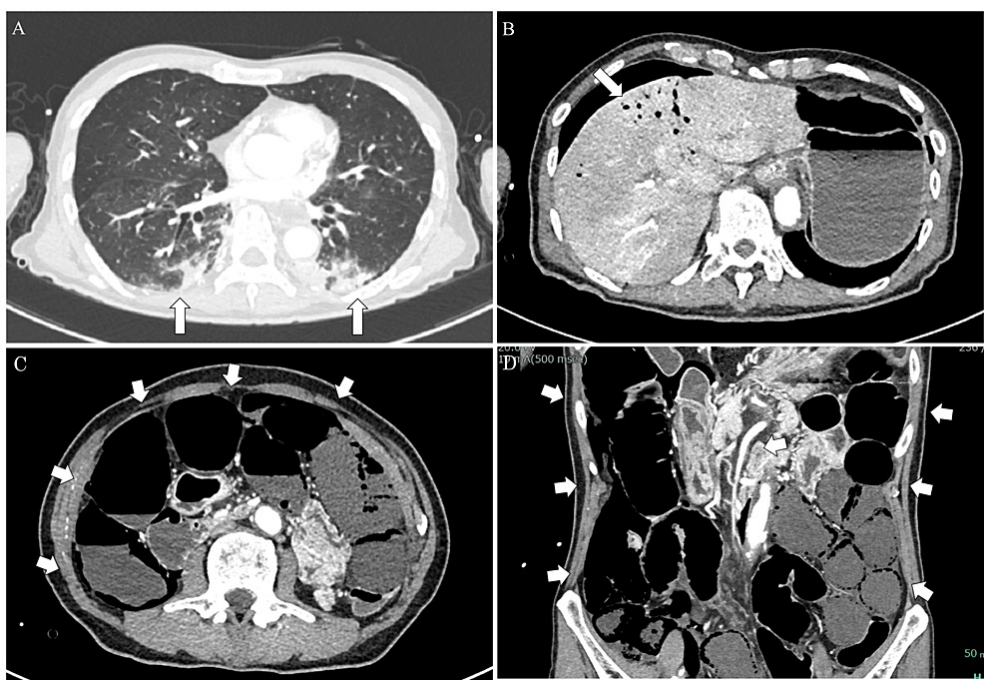
Contrast-enhanced CT image at the time of NOMI onset. (A) Patchy infiltrates are observed in the bilateral lungs. (B) Intrahepatic portal vein gas is found in the liver. (C) Extensive intestinal emphysema associated with intestinal necrosis is observed. (D) There is no occlusion of the superior mesenteric artery due to a thrombus or other reasons. CT: computed tomography, NOMI: nonocclusive mesenteric ischemia

Histopathological examination showed that both lesions were poorly differentiated SCC. Recurrent lesions on the right side of the neck tended to increase, and BRT with cetuximab and radiation therapy (2 Gy/day × 33 days) was started 43 days after neck dissection. On the 14th day after the start of BRT, the cervical lesion tended to shrink and remained in a reduced state. Fifteen days after the start of BRT, radiation mucositis grade 1 (Common Terminology Criteria for Adverse Events v4.0) appeared, and on the 23rd day, mucositis and dermatitis worsened to grade 2. On the 30th day, the mucositis worsened to grade 3, and tube feeding and oxycodone hydrochloride were started on the 31st day. On the 32nd day, nausea was observed, and on the 34th day, malaise developed, and intravenous infusion was started. On the 35th day, the patient showed abdominal distension and a decreased level of consciousness (Japan Coma Scare 100) with a respiratory rate of 20/min and heart rate of 120/min, and blood pressure decreased and became unmeasurable. Blood tests showed a leukocyte count of 9100/µL, C-reactive protein of 17.17 mg/dL, and platelet count of 94,000/µL. Procalcitonin, endotoxin, and beta-D-glucan were all positive, and blood gas analysis showed metabolic acidosis. Contrast-enhanced CT showed pneumonia in the bilateral lung fields, extensive intestinal necrosis and emphysema, and intraportal gas although the superior mesenteric artery was not obstructed (Figure 2A-2D).

**Figure 3:**
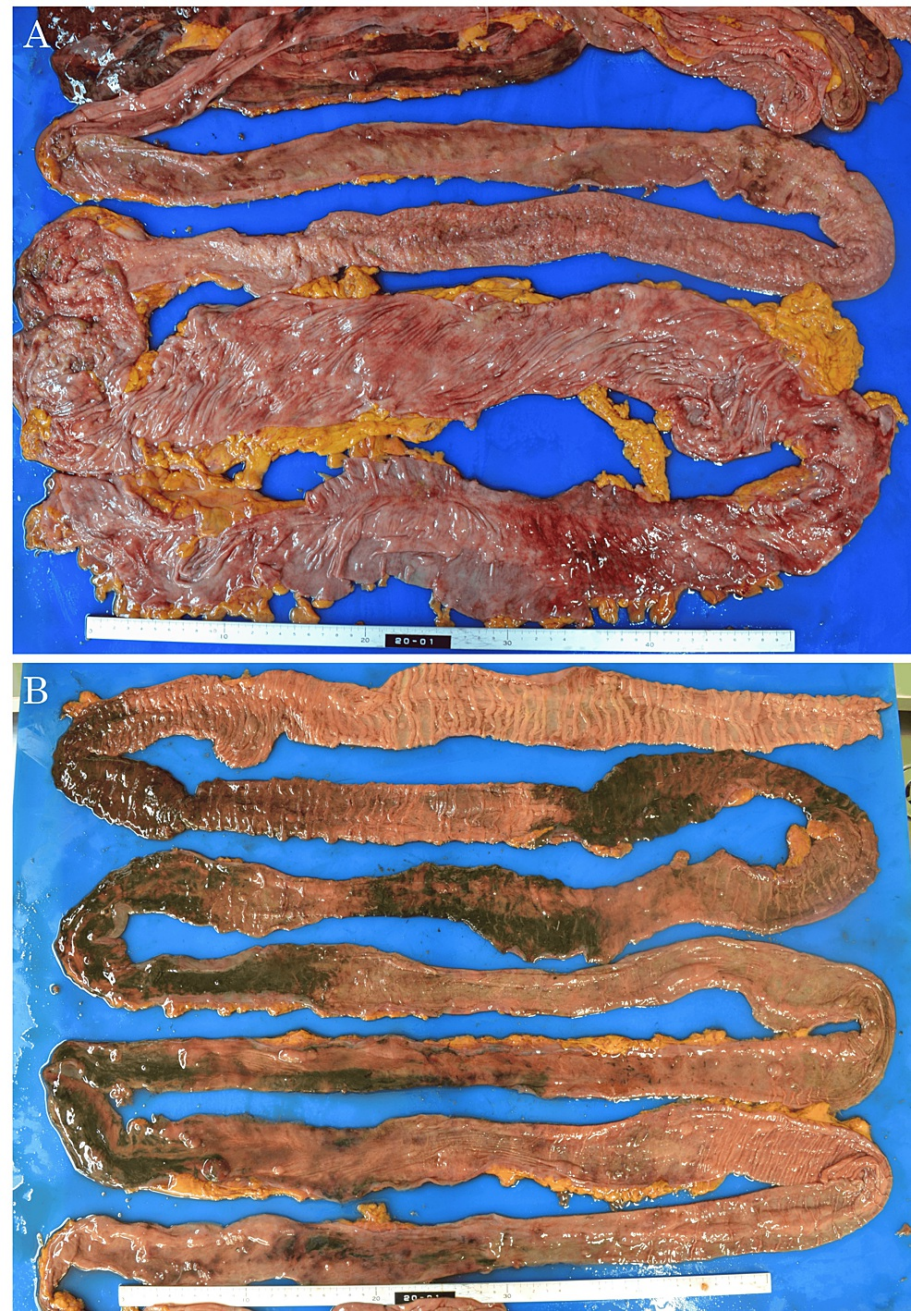
Intestinal photographs during autopsy. (A) Ischemic changes with redness are observed in the large intestine. (B) The small intestine shows necrosis with a speckled pattern of over approximately 3 m.

**Figure 4:**
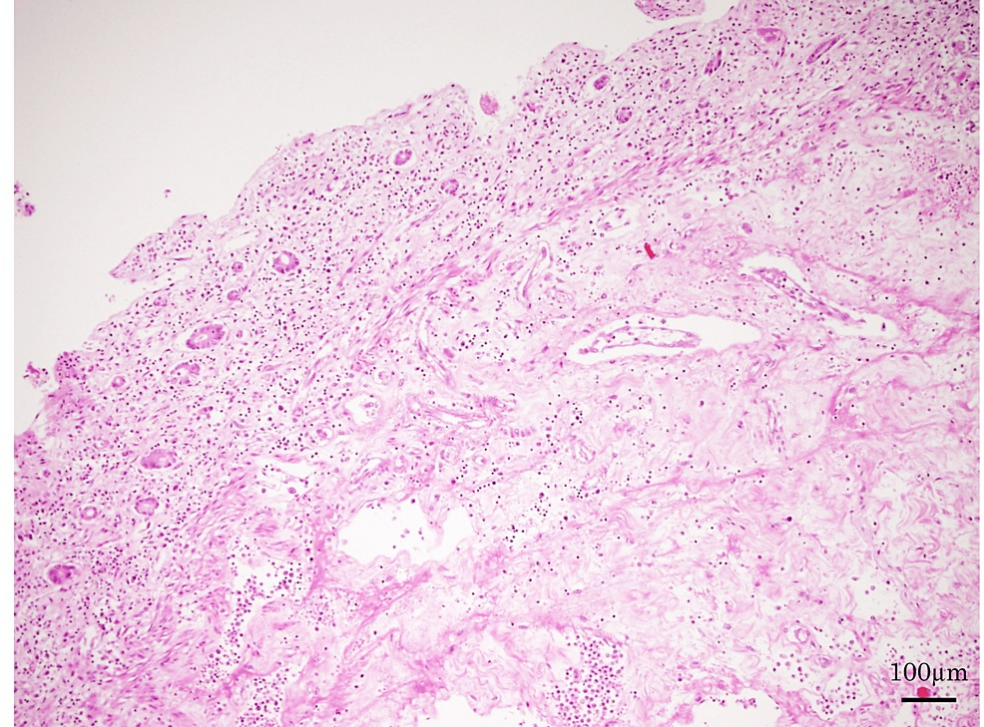
Histopathological image of the intestine during autopsy (hematoxylin and eosin stain). Total necrosis from mucosa to intrinsic muscular layer was observed (×100).

The patient was diagnosed with septic shock due to nonocclusive intestinal ischemia and was admitted to the intensive care unit. Meropenem hydrate was started, and circulatory agonists were administered, but his general condition did not improve, and he died on the same day. Pathological autopsy findings revealed that the recurrent site on the right side of the neck had responded well to treatment although some cancer cells remained. Gram-positive cocci and *Candida* were found in the lower lobes of both lungs, and severe aspiration pneumonia was observed. The large bowel showed mucosal necrosis with ischemic changes and redness, and the small intestine was necrotic in a speckled pattern of over 3 m (Figure 3A, 3B), with the histopathological examination showing complete necrosis (Figure 4).

No occlusion of the celiac artery or superior and inferior mesenteric arteries was observed, and the patient was diagnosed with intestinal necrosis due to nonocclusive intestinal ischemia. In addition, the liver was in a state of shock with necrosis around the central veins, and the heart showed evidence of infective endocarditis with verrucae on the aortic valve. The cause of death was identified as intestinal necrosis due to NOMI, severe aspiration pneumonia, and infective endocarditis, leading to septic shock.

## Discussion

NOMI is characterized by discontinuous ischemia and necrosis of the intestinal tract without organic occlusion of the main mesenteric vessels [1]. The causes of this disease include mesenteric artery spasms, atherosclerotic stenosis of the mesenteric artery, hypoxemia of the intestine, and ischemia/reperfusion injury; among these, mesenteric artery spasms are considered the most important [2]. The underlying diseases or triggering factors for this disease include heart failure, shock, digitalis poisoning, sepsis, gastrointestinal bleeding, dehydration, and hemodialysis [3-5]. The most common symptom of NOMI is abdominal pain, and the onset time is unclear. The degree and location of abdominal pain are not consistent with those in the case of vascular occlusive intestinal ischemia/necrosis [6]. Other symptoms include abdominal distension, melena, vomiting, and fever, but all these symptoms are nonspecific, and there are few objective findings in the early stages of the disease. Therefore, by the time the clinical findings are complete, the disease progresses, and the prognosis is extremely poor because of the possibility of shock and multiple organ failure [7]. In our patient, nausea occurred three days before the onset of shock, but opioids had been started the day before, and nausea was deemed an adverse event induced by opioids. When abdominal distension appeared, the patient’s condition worsened, and he was in shock. Conventionally, angiography is useful in the diagnosis of NOMI, and specific findings, such as narrowing of the superior mesenteric artery and poor angiography in the intestinal wall, have been reported. However, only a limited number of facilities can immediately perform angiography in cases of suspected NOMI. It is often not performed because of the poor general condition of patients and the complexity and invasiveness of the disease [8,9]. Recently, several studies have reported that multi-detector row CT (MDCT), which can noninvasively evaluate intestinal ischemia, is useful in developing treatment strategies for NOMI [9,10].

Kammerer et al. have reported that evaluating the diameter of the superior mesenteric artery using MDCT was comparable to angiography in terms of diagnostic performance for NOMI [10]. In our case, the patient was in shock when we suspected NOMI, and angiography was difficult to perform, so we diagnosed NOMI using contrast-enhanced CT. Contrast-enhanced CT is essential in cases of suspected NOMI when angiography cannot be performed. If NOMI is diagnosed early, it can be corrected by improving unstable circulatory dynamics that cause vasospasm and hypoxemia in cases without necrotizing findings or by conservative treatment, such as continuous infusion of prostaglandin E or papaverine hydrochloride [11]. However, in a state of intestinal necrosis, cardiotoxic substances are released from the necrotic epithelium, causing a vicious cycle in circulatory dynamics and irreversible shock, and early resection of the necrotic intestine is required [12]. As prognostic factors for NOMI, preoperative shock, the extent of intestinal necrosis, low platelet count, and severe acidosis have a poor prognosis [13].

In our case, the patient was in shock, and his condition did not improve even after the administration of circulatory agonists and had several poor prognostic factors, such as extensive necrosis of the small intestine and low platelet count, which would have made it difficult to save his life even if surgery could have been performed.

The number of cases of NOMI after chemotherapy is not high [14-18]. Among them, seven patients have developed NOMI after chemotherapy for head and neck cancer, and most cases of NOMI developed after docetaxel, cisplatin, and fluorouracil (TPF) therapy [15-18] (Table 1).

**Table 1:** Reported cases of NOMI during cancer treatment for head and neck cancer NOMI: nonocclusive mesenteric ischemia, CDDP: cisplatin, 5-FU: 5-fluorouraci, DOC: docetaxel, RT: radiotherapy

Author	Year	Age	Sex	Location	Cancer treatment	Treatment of NOMI	Outcome of NOMI
Wada et al. [15]	2017	74	Male	Nasopharynx	CDDP, 5-FU, DOC	Surgery	Alive
Tanaka et al. [16]	2018	74	Male	Nasopharynx	CDDP, 5-FU, DOC	Surgery	Alive
Yoshida et al. [17]	2020	63	Female	Maxillary sinus	CDDP, 5-FU, DOC	Surgery	Alive
Yoshida et al. [17]	2020	71	Male	Larynx	CDDP, 5-FU, DOC	Surgery	Alive
Nagano et al. [18]	2021	74	Male	Oropharynx	Oral S-1	Surgery	Alive
Nagano et al. [18]	2021	68	Male	Hypopharynx	CDDP, 5-FU, DOC	Conservative treatment	Deceased
Nagano et al. [18]	2021	82	Male	Hypopharynx	RT	Surgery	Alive
Our patient	2020	73	Male	Tongue	Cetuximab, RT	Conservative treatment	Deceased

The pathogenesis of NOMI caused by TPF therapy (docetaxel, cisplatin, and fluorouracil) is speculated to be due to the pharmacological effects of taxanes, such as mucosal damage caused by the mitotic proliferation of epithelial components and vascular damage caused by the inhibition of smooth muscle cell proliferation, migration, and neointimal accumulation, in addition to increased intestinal pressure caused by intestinal inflammation due to 5-fluorouracil and constipation [18,19]. Cetuximab, an IgG1 chimeric antibody that specifically binds to the target molecule epidermal growth factor receptor (EGFR), enhances the cytotoxic effects of radiation therapy in squamous cell carcinoma when used in combination [20]. In our patient, NOMI occurred during BRT, but no studies have reported NOMI after cetuximab treatment in other cancer types. The detailed mechanism of NOMI in our patient is unknown, but we believe that it may have been one of the rare adverse events that occur during concomitant radiation chemotherapy for head and neck cancer, rather than a sole effect of cetuximab.

We hypothesized that dysphagia due to severe mucositis caused by BRT led to aspiration pneumonia, resulting in dehydration, chronic renal failure, and sepsis, which decreased the circulating blood volume and caused intestinal ischemia due to peripheral mesenteric artery spasms, leading to the development of NOMI. To avoid the development of NOMI during BRT for head and neck cancer, preventing aspiration pneumonia through oral care and preventing dehydration through early tube feeding is important. Furthermore, if the patient complains of abdominal symptoms, such as abdominal pain or vomiting, performing early CT or consulting with a gastroenterologist without hesitation and discussing how to respond is crucial.

## Conclusions

We report a rare occurrence of NOMI during BRT with cetuximab for cervical lymph node metastasis of tongue cancer. Despite the absence of organic obstruction, NOMI resulted in mesenteric ischemia and extensive intestinal necrosis, ultimately leading to septic shock and the patient's demise. This emphasizes the need for heightened vigilance during similar therapeutic interventions and prompts further exploration of preventive measures for NOMI in tongue cancer management.
